# The effects of simulation-based education on undergraduate nursing students' competences: a multicenter randomized controlled trial

**DOI:** 10.1186/s12912-024-02069-7

**Published:** 2024-06-17

**Authors:** Lai Kun Tong, Yue Yi Li, Mio Leng Au, Wai I. Ng, Si Chen Wang, Yongbing Liu, Yi Shen, Liqiang Zhong, Xichenhui Qiu

**Affiliations:** 1 Kiang Wu Nursing College of Macau, Edifício do Instituto de Enfermagem Kiang Wu de Macau, Avenida do Hospital das Ilhas no.447, Coloane, RAEM, Macau SAR China; 2 School of Nursing, Yangzhou University, No.136, Jiangyang Middle Road, Hanjiang District, Yangzhou, Jiangsu Province China; 3 School of Nursing, Guangzhou Xinhua University, 19 Huamei Road, Tianhe District, Guangzhou, Guangdong Province China; 4 School of Nursing, Guangzhou Medical University, Dongfeng West Road, Yuexiu District, Guangzhou, Guangdong Province China; 5 School of Nursing, Shenzhen University, No. 3688, Nanhai Road, Nanshan District, Shenzhen, Guangdong Province China

**Keywords:** High-fidelity simulation, Computer-based simulation, High-fidelity simulation combined with computer-based simulation, Case study, Knowledge, Skill, Interprofessional collaboration, Critical thinking, Caring, Interest in learning

## Abstract

**Background:**

Education in nursing has noticed a positive effect of simulation-based education. There are many studies available on the effects of simulation-based education, but most of those involve a single institution, nonrandomized controlled trials, small sample sizes and subjective evaluations of the effects. The purpose of this multicenter randomized controlled trial was to evaluate the effects of high-fidelity simulation, computer-based simulation, high-fidelity simulation combined with computer-based simulation, and case study on undergraduate nursing students.

**Methods:**

A total of 270 nursing students were recruited from five universities in China. Participants were randomly divided into four groups at each institution: the high-fidelity simulation group, the computer-based simulation group, the high-fidelity simulation combined with computer-based simulation group, and the case study group. Finally, 239 participants completed the intervention and evaluation, with 58, 67, 57, and 57 participants in each group. The data were collected at three stages: before the intervention, immediately after the intervention, and three months after the intervention.

**Results:**

The demographic data and baseline evaluation indices did not significantly differ among the four groups. A statistically significant difference was not observed between the four methods for improving knowledge, interprofessional collaboration, critical thinking, caring, or interest in learning. While skill improvement differed significantly among the different groups after the intervention (*p* = 0.020), after three months, no difference was observed (*p* = 0.139). The improvement in skill in the computer-based simulation group was significantly lower at the end of the intervention than that in the high-fidelity simulation group (*p* = 0.048) or the high-fidelity simulation combined with computer-based simulation group (*p* = 0.020).

**Conclusions:**

Nursing students benefit equally from four methods in cultivating their knowledge, interprofessional collaboration, critical thinking, caring, and interest in learning both immediately and over time. High-fidelity simulation and high-fidelity simulation combined with computer-based simulation improve skill more effectively than computer-based simulation in the short term. Nursing educators can select the most suitable teaching method to achieve the intended learning outcomes depending on the specific circumstances.

**Trial registration:**

This clinical trial was registered at the Chinese Clinical Trial Registry (clinical trial number: ChiCTR2400084880, date of the registration: 27/05/2024).

## Introduction

There are many challenges nursing students face in the clinical setting because of the gap between theory and practice, the lack of resources, and unfamiliarity with the medical environment [1]. Nursing education needs an innovative teaching method that is more closely related to the clinical environment. Simulation-based education is an effective teaching method for nursing students [2]. It provides students with an immersive clinical environment for practicing skills and gaining experience in a safe, controlled setting [3]. This educational approach not only supports the development of various competencies [2, 4], including knowledge, skill, interprofessional collaboration, critical thinking, caring, and interest in learning, but also enables students to apply learned concepts to complex and challenging situations [5].

Manikin-based and computer-based simulations are commonly employed simulators in nursing education. Manikin-based simulation involves the use of a manikin to mimic a patient’s characteristics, such as heart and lung sounds [6]. Computer-based simulation involves the modeling of real-life processes solely using computers, usually with a keyboard and monitor as inputs and outputs [6]. According to a recent meta-analysis, manikin-based simulation improves nursing students' knowledge acquisition more than computer-based simulation does, but there are no significant differences in confidence or satisfaction with learning [4].

Based on the level of fidelity, manikin-based simulation can be categorized as low, medium, or high fidelity [7]. High-fidelity simulation has become increasingly popular since it replaces part of clinical placement without compromising nursing student quality [8]. Compared to other teaching methods, high-fidelity simulation is associated with elevated equipment and labor costs [9]. To enhance cost-effectiveness, it is imperative to maximize the impact of high-fidelity simulation. To improve learning outcomes, mixed learning has gained popularity across higher education in recent years [10]. The most widely used mixed learning method for simulation education in the nursing field is high-fidelity simulation combined with computer-based simulation. There have been only a few studies on the effect of high-fidelity simulation combined with computer-based simulation on nursing students, and these are either pre-post comparison studies without control groups [11] or quasi-experimental studies without randomization [12]. To obtain a better grasp of the effects of combining high-fidelity simulation and computer-based simulation, a randomized controlled trial is needed.

In addition to enhancing effectiveness, optimizing cost-effectiveness can be achieved by implementing cost reduction measures. Case study, which eliminates the need for additional equipment, offers a relatively low-cost alternative. A traditional case study provides all pertinent information, whereas an unfolding case study purposefully leaves out information [13]. It has been shown that unfolding case study fosters critical thinking in students more effectively than traditional case studies [14]. Despite being regarded as an innovative and inexpensive teaching method, there is little research comparing unfolding case study with other simulation-based teaching methods. To address this knowledge gap, further study is necessary.

An umbrella review highlights that the existing literature on the learning outcomes of simulation-based education predominantly emphasizes knowledge and skills, while conferring limited focus on other core competencies, such as interprofessional collaboration and caring [15]. Therefore, future research should evaluate various learning outcome indicators.

This multicenter randomized controlled trial aimed to assess the effectiveness of high-fidelity simulation, computer-based simulation, high-fidelity simulation combined with computer-based simulation, and case study on nursing students’ knowledge, skill, interprofessional collaboration, critical thinking, caring, and interest in learning.

## Method

### Study design

A multicenter randomized controlled trial was conducted between March 2022 and May 2023 in China. The study conforms to the CONSORT guidelines. This clinical trial was registered at the Chinese Clinical Trial Registry (clinical trial number: ChiCTR2400084880, date of the registration: 27/05/2024).

### Participants and setting

Participants were recruited from five universities in China, two of which were private and three of which were public. Among the five universities, four were equipped with two high-fidelity simulation laboratories. Specifically, three universities had laboratories simulating intensive care unit wards and delivery rooms, while the remaining university had two laboratories simulating general wards. Additionally, one university possessed a high-fidelity simulation laboratory specifically designed to simulate a general ward setting. Three universities utilized Laerdal patient simulators in their laboratories, while the other two universities employed Gaumard patient simulators.

A recruitment poster with the time and location of the project promotion was posted on the school bulletin board. The research team provided a briefing to students at the designated time and location indicated on the poster, affording them the opportunity to inquire about and enhance their understanding of the project.

The study mandated that participants fulfill the following criteria: 1) enroll in a nursing undergraduate program; 2) have full-time student status; 3) complete courses in Anatomy and Physiology, Pathophysiology, Pharmacology, Health Assessment, Basic Nursing, and Medical and Surgical Nursing (Respiratory System); 4) have proficiency in reading and writing Chinese; and 5) participate voluntarily. Those who met the following criteria were excluded: 1) had a degree or diploma and 2) took the course again.

The sample size was calculated through the use of G*Power 3.1, which was based on F tests (ANOVA: Repeated measures, between factors). Several assumptions were taken into consideration, including a 5% level of significance, 80% power, four groups, three measurements, and a 0.50 correlation between pre- and postintervention time points. Compared to other teaching methods, high-fidelity simulation exhibited a medium effect size (d = 0.49 for knowledge, d = 0.50 for performance) [16]. The calculation employed a conservative approach, accommodating a small yet clinically significant effect size (0.25), thereby bolstering the reliability and validity of the findings. Based on these assumptions, the total sample size required was determined to be 124, with each group requiring 31 participants.

### Randomization and blinding

Due to inconsistent teaching schedules at the five universities involved in the study, the participants were divided into four groups at each institution: the high-fidelity simulation group, the computer-based simulation group, the high-fidelity simulation combined with computer-based simulation group, and the case study group. Participant grouping was carried out by study team members who were not involved in the intervention or evaluation. The participants were each assigned a random nonduplicate number between zero and 100 using Microsoft Excel. The random numbers/participants were divided into four groups based on quartiles: the lower quarter, the lower quarter to a half, the half to three-fourths, and the upper quarter were assigned to the high-fidelity simulation group, the computer-based simulation group, the high-fidelity simulation combined with computer-based simulation group, and the case study group, respectively. It was not possible to implement participant blinding because the four teaching methods differed significantly, while effect evaluation and data analysis were conducted in a blinded manner. Each participant was assigned a unique identifier to maintain anonymity throughout the study.

### Procedures

#### Baseline test

Baseline testing started after participant recruitment had ended, so the timing of the study varied between universities. The baseline test items were the same for all participants and included general characteristics, knowledge, skills, interprofessional collaboration, critical thinking, caring, and interest in learning. The evaluation of skills was conducted by trained assessors, whereas a non-face-to-face online survey was utilized for the assessment of others.

#### Intervention

The four groups were taught with three scenarios covering the three different cases, in the following order: asthma worsening, drug allergy, and ventricular fibrillation. These three cases represent commonly encountered scenarios necessitating emergency treatment. It is anticipated that by means of training, students can enhance their aptitude to effectively handle emergency situations within clinical settings. It is vital that the case used in simulation-based education is valid so that its effectiveness can be enhanced [17]. The cases used in this study were from vSim® for Nursing | Lippincott Nursing Education, which was developed by Wolters Kluwer Health (Lippincott), Laerdal Medical, and the National League for Nursing. Hence, the validity of the cases can be assured. Participants received all the materials, including learning outcomes, theoretical learning materials, and case materials (medical history and nursing document), at least one day before teaching. All the teachers in charge of teaching participated in the meeting to discuss the lesson plans to reach a consensus on the lesson plans. The lesson plans were written by three members of the research team and revised according to the feedback. Table 1 shows the teaching experience of each case in the different intervention groups. The instructors involved had at least five years of teaching experience and a master's degree or higher.

**Table 1:** Intervention descriptions

Group	Time	Procedures and Component	Form
High-fidelity simulation group	70	1) Briefing (10 min): Participants were introduced to the learning outcomes, laboratory environment, and patient conditions 2) Simulation (15 min): Participants practiced in a simulated environment with SimMan® 3G. Participants were divided into active participants (three) and observers (two to three). Among the active participants, one played the role of a senior nurse, and the other two played the roles of junior nurses 3) Debriefing (45 min): The instructor and participants re-examined the simulation experience	Group face-to-face instruction between an instructor and participants (between four and six participants) at a mutually agreed upon time and place. The instructor played the role of the doctor
Computer-based simulation group	60	vSim® for Nursing was used. Participants were instructed to follow the vSim format 1) Presimulation quiz: Participants received feedback after answering the questions 2) Simulation (less than 30 min): Participants received individualized feedback after completing the vSim scenario 3) Postsimulation quiz: Participants received feedback after answering the questions	It was a self-paced online practice that participants completed by themselves before the deadline
High-fidelity simulation combined with computer-based simulation group	130	Participants performed computer simulation followed by high-fidelity simulation. For the computer-based simulation, the intervention protocol was the same as for the computer-based simulation group, while for the high-fidelity simulation, it was the same as for the high-fidelity simulation group	For the computer-based simulation, the intervention form was the same as for the computer-based simulation group, while for the high-fidelity simulation, it was the same as for the high-fidelity simulation group
Case study group	60	Briefing (10 min): Participants were introduced to the learning outcomes, and patient conditions Discussion (35 min): The discussion was prompted by written questions about the scenario. In response to the participants’ request, the instructor provided the patient's additional clinical data Debriefing (15 min): The instructor and participants re-examined the experience	Face-to-face instruction between an instructor and participants at a mutually agreed upon time and place. The instructor played the role of the doctor

#### Posttest and follow-up test

The posttest was conducted within one week of the intervention using the same items as those used in the baseline test. The follow-up test was administered after three months of the intervention.

### Measures

#### General characteristics

The general characteristics of the participants included gender, age, and previous semester grade.

#### Knowledge

This was measured by five multiple-choice items developed for this study. The items were derived from the National Nurse Licensing Examination [18]. The maximum score was five, with one awarded for each correct answer. The questionnaire exhibited high content validity (CVI = 1.00) and good reliability (Kuder-Richardson 20 = 0.746).

#### Skill

The Creighton Competency Evaluation Instrument (CCEI) is designed to assess clinical skills in a simulated environment by measuring 23 general nursing behaviors. This tool was originally developed by Todd et al. [19] and subsequently modified by Hayden et al. [20]. The Chinese version of the CCEI has good reliability (Cronbach’s α = 0.94) and validity (CVI = 0.98) [21]. The CCEI was scored by nurses with master’s degrees who were trained by the research team and blinded to the intervention information. A dedicated person was assigned to handle the rating for each university, and the raters did not rotate among the participants. The Kendall's W coefficient for the raters' measures was calculated to be 0.832, indicating a high level of interrater agreement and reliability. All participants were tested using a high-fidelity simulator, with each test lasting ten minutes. The skills test without debriefing employed a single-person format, and the nursing procedures did not rely on laboratory results, so the items "Delegates Appropriately," "Reflects on Clinical Experience," "Interprets Lab Results," and "Reflects on Potential Hazards and Errors" were excluded from the assessment. The total score ranged from 0–19 and a higher score indicated a higher level of skill.

#### Interprofessional collaboration

The Assessment of the Interprofessional Team Collaboration Scale for Students (AITCS-II Student) was used to assess interprofessional collaboration. It consists of 17 items rated on a 5-point Likert scale (1 = never, 5 = always), for a total score ranging from 17 to 85 [22]. The Chinese version of the AITCS-II has good reliability (Cronbach’s α = 0.961) and validity [23].

#### Critical thinking

Critical thinking was measured by Yoon's Critical Thinking Disposition Scale (YCTD). It is a five-point Likert scale with values ranging from 1 to 5, resulting in a total score ranging from 27 to 135 [24]. Higher scores on this scale indicate greater critical thinking ability. The YCTD has good reliability (Cronbach’s α = 0.948) and validity when applied to Chinese nursing students [25].

#### Caring

Caring was assessed using the Caring Dimensions Inventory (CDI), which employs a five-point Likert scale ranging from 25 to 125 [26]. Higher scores on the CDI indicate a greater level of caring. The Chinese version of the CDI exhibited good reliability (Cronbach’s α = 0.97) and validity [27].

#### Interest in learning

The Study Interest Questionnaire (SIQ) was used to assess interest in learning. The SIQ is a four-point Likert scale ranging from 18 to 72, where a higher total score indicates a greater degree of interest in the field of study [28]. The SIQ has good reliability (Cronbach’s α = 0.90) and validity when applied to Chinese nursing students [29].

### Ethical considerations

The institution of the first author granted ethical approval (ethical approval number: REC-2021.801). Written informed consent was obtained from all participants. Participants were permitted to withdraw for any reason at any time without penalty. Guidelines emphasizing safety measures and precautions during the intervention were provided to participants, and study coordinators closely monitored laboratory and simulation sessions to address concerns or potential harm promptly.

### Data analysis

Descriptive statistics were used to describe the participant characteristics and baseline characteristics. Continuous variables are presented as the mean and standard deviation, while categorical variables are presented as frequencies and percentages. According to the Quantile–Quantile Plot, the data exhibited an approximately normal distribution. Furthermore, Levene's test indicated equal variances for the variables of knowledge, skill, interprofessional collaboration, critical thinking, caring, and interest in learning, with p-values of 0.171, 0.249, 0.986, 0.634, 0.992, and 0.407, respectively. The baseline characteristics of the four groups were compared using one-way analysis of variance. The indicators of knowledge, skill, interprofessional collaboration, critical thinking, caring, and interest in learning were assessed at baseline, immediately after the intervention, and three months postintervention. Changes in these indicators from baseline were calculated for both the postintervention and three-month follow-up periods. The changes among the four groups were compared using one-way analysis of variance. Cohen's d effect sizes were computed for the between-group comparisons (small effect size = 0.2; medium effect size = 0.5; large effect size = 0.8). Missing data were treated as missing without imputation. The data analysis was conducted using jamovi 2.3.28 (https://www.jamovi.org/). Jamovi was developed on the foundation of the R programming language, and is recognized for its user-friendly interface. The threshold for statistical significance was established at a two-sided *p* < 0.05.

## Results

### Participants

**Fig. 1:**
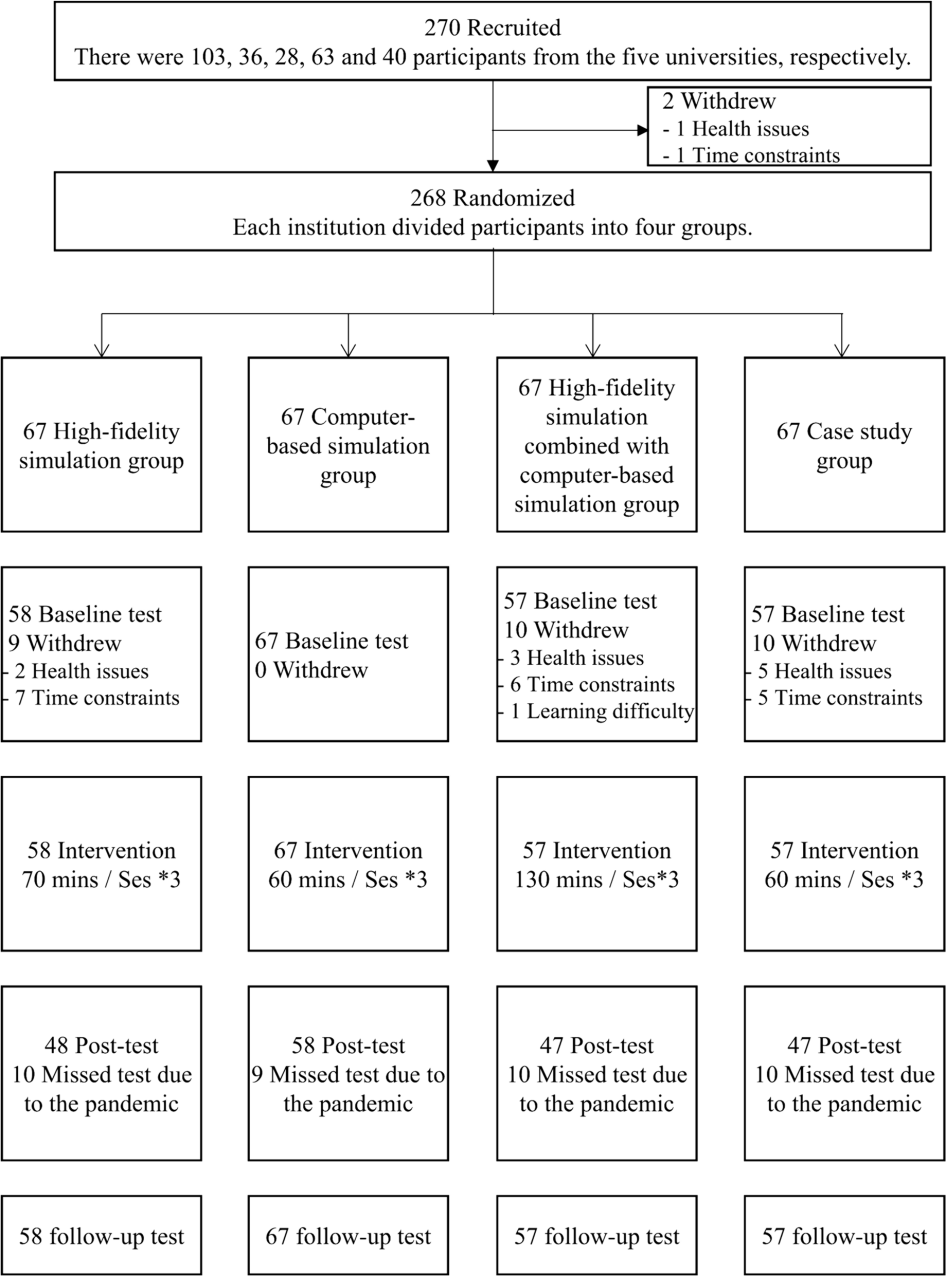
Study subject disposition flow chart

A total of 270 participants were initially recruited from five universities for this study. However, an attrition rate of 11.5% was observed, resulting in 31 participants discontinuing their involvement. Consequently, the final analysis included data from 239 participants who successfully completed the intervention and remained in the study. Specifically, there were 58 participants in the high-fidelity simulation group, 67 in the computer-based simulation group, 57 in the high-fidelity simulation combined with computer-based simulation group, and 57 in the case study group (Fig. 1). The participant demographics and baseline characteristics are displayed in Table 2, and no significant differences were observed in these variables.

**Table 2:** Participant demographics and baseline characteristics

	High-fidelity simulation group (*n* = 58)	Computer-based simulation group (*n* = 67)	High-fidelity simulation combined with computer-based simulation group (*n* = 57)	Case study group (*n* = 57)	F/χ^2^	*p*
Gender ^a^	9 (49)	13 (54)	11 (46)	9 (48)	0.566	0.904
Age	21.2 ± 1.7	21.0 ± 0.9	21.1 ± 1.9	20.9 ± 1.4	0.494	0.687
Academic performance	72.1 ± 10.5	70.8 ± 16.0	71.1 ± 11.4	69.6 ± 15.6	0.357	0.784
Knowledge	2.7 ± 1.3	2.5 ± 1.1	2.7 ± 1.4	2.8 ± 1.2	0.672	0.571
Skill	4.8 ± 3.1	5.5 ± 3.1	6.0 ± 2.5	5.4 ± 3.0	1.564	0.201
Interprofessional collaboration	71.0 ± 8.8	68.7 ± 9.2	69.7 ± 9.1	69.4 ± 8.8	0.688	0.561
Critical thinking	97.5 ± 12.3	99.6 ± 8.5	99.8 ± 8.1	101.7 ± 10.6	1.296	0.279
Caring	106.0 ± 11.2	104.4 ± 11.4	106 ± 11.2	107.1 ± 10.7	0.623	0.601
Interest in learning	49.0 ± 6.3	51.0 ± 6.1	50.7 ± 7.2	50.4 ± 6.3	1.226	0.303

Note: a The data are presented as male (female) and are the outcome of a chi-square test

### Efficacy outcomes

#### Knowledge

**Fig. 2:**
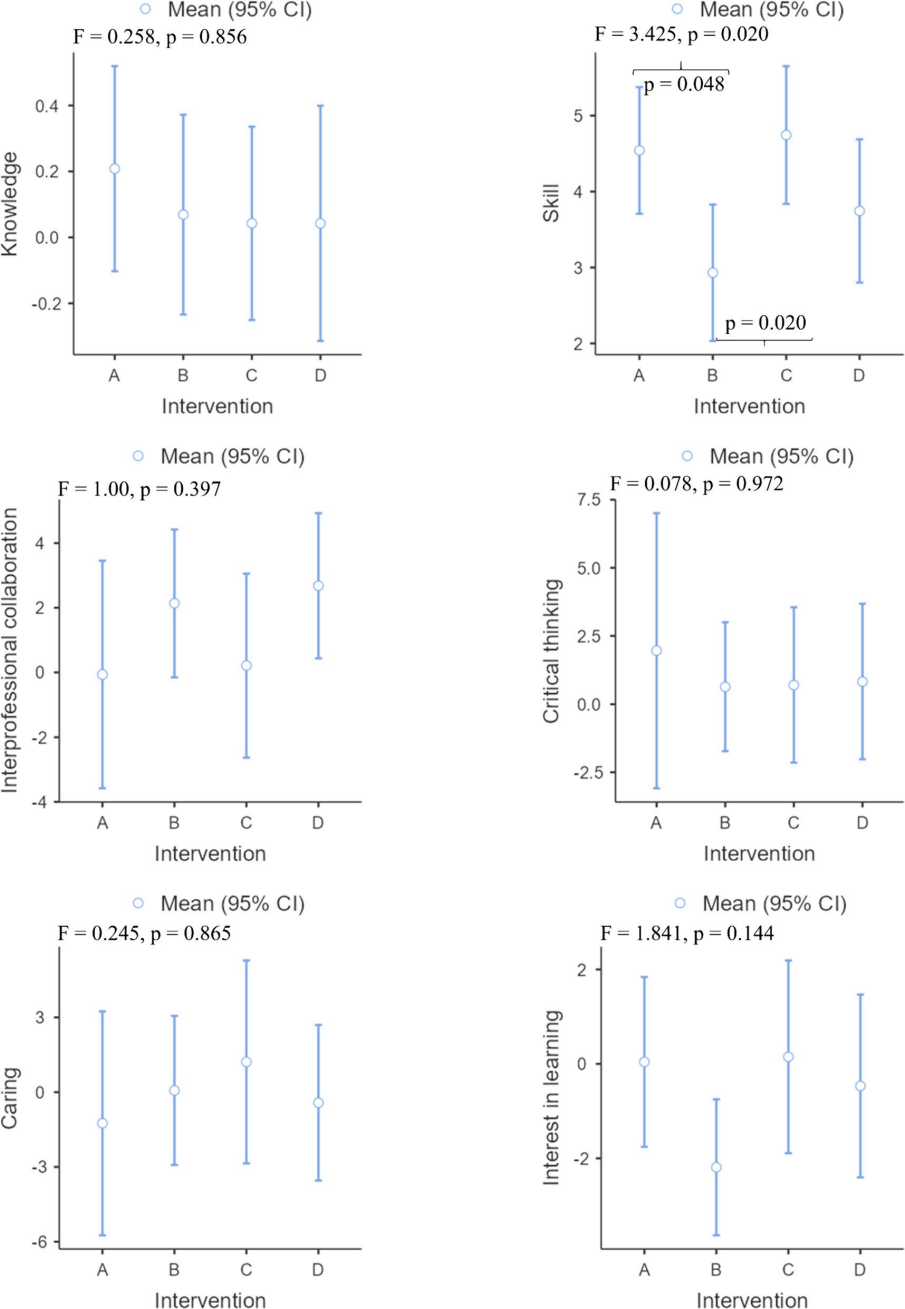
Changes in all effectiveness outcomes at post intervention. Note: **A** High-fidelity simulation group; **B** Computer-based simulation group; **C** High-fidelity simulation combined with computer-based simulation group; **D** Case study group

**Fig. 3:**
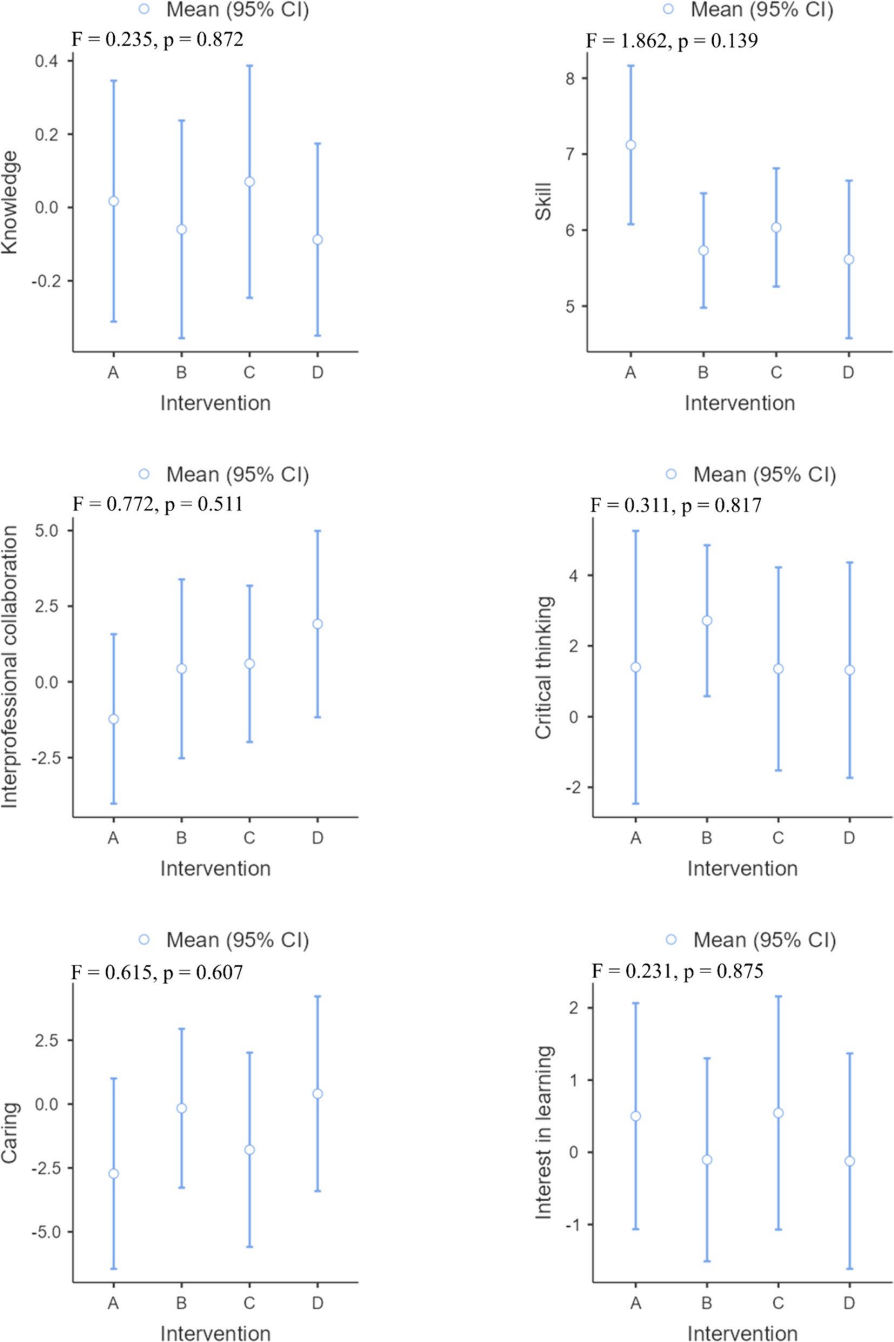
Changes in all effectiveness outcomes at three months of intervention. Note: **A **High-fidelity simulation group;  **B**  Computer-based simulation group;  **C**  High-fidelity simulation combined with computer-based simulation group;  **D**  Case study group

All the intervention groups showed improvements in knowledge after the intervention, with the high-fidelity simulation group showing the greatest improvement (Fig. 2). However, there were no significant differences in knowledge improvement among the groups (p = 0.856). The computer-based simulation group and case study group experienced a decrease in knowledge compared to baseline three months after the intervention, while the other groups showed an increase in knowledge. The high-fidelity simulation combined with computer-based simulation group performed best (Fig. 3), but no significant differences were observed (p = 0.872). The effect sizes between groups were found to be small, both immediately after the intervention and at the three-month follow-up (Table 3).

**Table 3:** Between-group Cohen d effect sizes

	High-fidelity simulation vs Computer-based simulation	High-fidelity simulation vs High-fidelity simulation combined with computer-based simulation	High-fidelity simulation vs Case study	Computer-based simulation vs High-fidelity simulation combined with computer-based simulation	Computer-based simulation vs Case study	High-fidelity simulation combined with computer-based simulation vs Case study
Post intervention						
Knowledge	0.13	0.16	0.14	0.02	0.02	0.00
Skill	0.51	0.07	0.26	0.56	0.25	0.32
Interprofessional collaboration	0.21	0.03	0.27	0.21	0.07	0.28
Critical thinking	0.10	0.09	0.08	0.01	0.02	0.01
Caring	0.10	0.17	0.06	0.09	0.04	0.13
Interest in learning	0.38	0.02	0.08	0.37	0.28	0.09
After three months of the intervention						
Knowledge	0.06	0.04	0.09	0.11	0.03	0.14
Skill	0.39	0.31	0.38	0.10	0.03	0.12
Interprofessional collaboration	0.15	0.18	0.29	0.01	0.12	0.12
Critical thinking	0.11	0.00	0.01	0.14	0.14	0.00
Caring	0.19	0.07	0.22	0.12	0.04	0.15
Interest in learning	0.10	0.01	0.11	0.11	0.00	0.11

#### Skill

The different intervention groups showed improvements in skills after the intervention and three months after the intervention. The high-fidelity simulation combined with computer-based simulation group showed the greatest improvement after the intervention (Fig. 2), while the greatest improvement was observed in the high-fidelity simulation group three months after the intervention (Fig. 3). There was a significant difference in the improvement in skills among the different groups after the intervention (*p* = 0.020). Specifically, the improvement observed in the computer-based simulation group was significantly lower than that in both the high-fidelity simulation group (*p* = 0.048) and the high-fidelity simulation combined with computer-based simulation group (*p* = 0.020). However, three months after the intervention, there was no statistically significant difference in skill improvement among the groups (*p* = 0.139). Except for the between-group effect sizes of the high-fidelity simulation group compared to the computer-based simulation group (Cohen d = 0.51) and the computer-based simulation group compared to the high-fidelity simulation combined with computer-based simulation group (Cohen d = 0.56), the effects were found to be medium after the intervention, while the other between-group effect sizes were small both after the intervention and three months after the intervention (Table 3).

#### Interprofessional collaboration

In all intervention groups except for the high-fidelity simulation group, interprofessional collaboration improved after the intervention and three months after the intervention, with the case study group (Figs. 2 and 3) demonstrating the greatest improvement. No significant difference was found between the intervention groups after or three months after the intervention in terms of changes in interprofessional collaboration. Both immediately following the intervention and three months later, the effect sizes between groups were small (Table 3).

#### Critical thinking

After the intervention and three months after the intervention, the critical thinking of all the intervention groups improved. Among them, the high-fidelity simulation group improved the most after the intervention (Fig. 2), while the computer-based simulation group improved the most three months after the intervention (Fig. 3). However, no statistically significant differences were observed in the improvement of critical thinking across the different groups. The between-group effect sizes of each group were small both after the intervention and three months after the intervention (Table 3).

#### Caring

Caring improved following the intervention in all intervention groups, with the exception of the high-fidelity simulation group and case study group (Fig. 2). However, no significant difference was observed between the intervention groups in terms of changes (*p* = 0.865). A decrease in caring was observed three months after the intervention in all intervention groups, except for the case study group (Fig. 3). Nevertheless, no statistically significant difference was detected between the intervention groups in terms of changes (p = 0.607). Both immediately following the intervention and three months later, the effect sizes between groups were small (Table 3).

#### Interest in learning

In terms of interest in learning, both the high-fidelity simulation group and the high-fidelity simulation combined with computer-based simulation group improved after the intervention or three months later. Among the groups, the high-fidelity simulation combined with computer-based simulation group improved the most after both the intervention and three months after the intervention (Figs. 2 and 3). However, no statistically significant difference was detected between the intervention groups in terms of changes either after the intervention (p = 0.144) or three months after the intervention (p = 0.875). Both immediately following the intervention and three months later, the effect sizes between groups were small (Table 3).

## Discussion

To our knowledge, this study is the first multicenter randomized controlled trial to explore the effects of different simulation teaching methods on nursing students' competence and the first study in which multiple different indicators were evaluated simultaneously. The indicators included both objectively assessed indicators of knowledge and skills and subjectively assessed indicators of interprofessional collaboration, critical thinking, caring, and interest in learning. This study assessed the immediate and long-term effects of the intervention by examining its immediate impact as well as its effects three months postintervention.

The results obtained from this study indicate that high-fidelity simulation, computer-based simulation, high-fidelity simulation combined with computer-based simulation, and case study could improve nursing students’ knowledge immediately after intervention. Furthermore, these four teaching methods exhibited comparable effectiveness in improving knowledge. The findings of this study contradict previous meta-analyses that showed that high-fidelity simulation improved nursing students' knowledge over other teaching techniques [2]. This discrepancy may be attributed to the inclusion of simulation teaching in the previous study alongside theoretical teaching [12], whereas the current study solely employed simulation teaching without incorporating theoretical instruction. Notably, three months following the intervention, computer-based simulation and case study did not result in knowledge retention. Conversely, high-fidelity simulation, particularly when combined with computer-based simulation, demonstrated knowledge retention, with the latter exhibiting superior performance in this regard. The realistic nature of the simulation provided students with a context in which to apply their knowledge, enhancing their understanding of key concepts [30]. High-fidelity simulation surpasses computer-based simulation and case study in terms of realism. When combined with computer-based simulation, this approach affords students the opportunity to practice their knowledge in a safe environment while also providing them with access to additional resources and learning opportunities [31]. Therefore, in this study, high-fidelity simulation combined with computer-based simulation proved to be the most effective at retaining knowledge.

Four simulation-based education strategies were found to be effective at acquiring and retaining skills by the students in this study. High-fidelity simulation combined with computer-based simulation was found to be more effective at acquiring skill than was using either method alone. This method combines the benefits of both teaching methods, providing students with a comprehensive learning experience that combines physical realism and virtual interactivity [32]. Hybrid simulation creates a seamless learning experience in which individuals can practice their skills in a simulated environment, receive immediate feedback, and then transfer those skills to real-world situations. This integration provides a seamless transition from theoretical knowledge to practical skills, making it easier for individuals to apply what they have learned and enhance their overall performance [33]. Hybrid simulation may seem to be an attractive option [34]; however, this study found that hybrid simulation had no advantage in terms of skill retention; rather, high-fidelity simulation performed best. More research is needed in the future to confirm the results of this study and the underlying reasons since previous studies have not compared hybrid simulation with high-fidelity simulation on skill retention.

The findings of this study reveal a noteworthy observation: interprofessional collaboration improved across all interventions, except for high-fidelity simulation. This finding diverges from prior studies that indicated high-fidelity simulation as a more effective method for enhancing students' interprofessional collaboration compared to traditional case study [35]. This discrepancy may be attributed to the use of an unfolding case study in the current study, wherein patient scenarios evolve unpredictably, thereby prompting students and team members to engage in heightened collaborative efforts to address evolving patient care challenges [36]. Interprofessional collaboration plays a crucial role in improving healthcare outcomes. Studies have shown that when healthcare professionals collaborate effectively, patients experience better outcomes, fewer errors, and shorter hospital stays [37]. While high-fidelity simulation has gained popularity as a training tool, according to the results of this study, its impact on interprofessional collaboration remains limited. There may be two reasons for this. First, high-fidelity simulation scenarios are often time constrained [38], which can hinder effective interprofessional collaboration. Each team member may prioritize their individual goals or tasks, making it difficult to achieve optimal teamwork and coordination. Second, interprofessional team members may not have worked together extensively, which can hinder their ability to collaborate effectively in a high-fidelity simulation setting. It takes time to build trust and rapport, which may not be readily available in a simulated environment [39]. Despite being assigned the roles of senior nurse or junior nurse, participants in the high-fidelity simulation group were provided with the opportunity to engage with peers at various levels and individuals from different professions, such as instructors assuming the role of doctors. However, the duration of the simulation section for this group was limited to only 10 min. In contrast, participants in the computer-based simulation group and case study group were allocated 30 min and 35 min, respectively. It is crucial for healthcare institutions and educators to critically evaluate their simulation-based training programs and incorporate key components that promote interprofessional collaboration [40].

This study revealed that four interventions effectively promoted students' critical thinking, and these effects lasted for three months after the interventions. Furthermore, high-fidelity simulation was most effective at improving critical thinking in the short term, whereas computer-based simulation was most effective at fostering long-term improvements. High-fidelity simulation involves creating a realistic and immersive environment that closely resembles a real-world scenario [41]. This approach affords individuals the opportunity to actively participate and immerse themselves in the simulated scenario, thereby enhancing their experiential understanding [3]. Computer-based simulation does not provide the same immediate and tangible experience as high-fidelity simulation. High-fidelity simulation commonly incorporates the utilization of medical devices and mannequins that closely resemble clinical scenarios, thereby affording students a more authentic and immersive learning encounter. Only 5% of students perceive computer-based simulation as a viable substitute for mannequin-based simulation within the curriculum [42]. As a result, high-fidelity simulation is highly effective in the short term, and a previous meta-analysis reported similar results [43]. However, computer-based simulation provides advantages for data collection and analysis that contribute to the long-term development of critical thinking skills. In the simulation, participants can record their actions, decisions, and results [3]. These data can be used to compare different strategies and approaches, allowing participants to reflect on their own critical thinking skills and identify areas for improvement. Furthermore, it is noteworthy that the four simulation teaching methods demonstrated the ability to enhance students' critical thinking. However, it is important to consider the substantial disparity in costs among these methods. Therefore, educators should carefully evaluate their available resources and opt for the most cost-effective approach to foster students' critical thinking.

This study found limited evidence that all four simulation teaching methods contribute to improve caring among students. High-fidelity simulation often focuses on technical skills rather than patient interaction or emotional sensitivity [44, 45]. Moreover, research has demonstrated that using mannequins in high-fidelity simulation leads some students to perceive them as separate from real-life patients [45]. This perception reduces students' concern for the consequences of their actions during the simulation [45], hindering empathy development and limiting the cultivation of their caring abilities [46]. Unlike high-fidelity simulation, which provides tactile experiences and simulates real-life interactions, computer-based simulation is characterized by the absence of human connections. This lack of physical proximity can hinder the development of caring behaviors such as nonverbal communication, empathy, and sympathy [47, 48]. Similarly, the absence of direct patient interaction is a notable drawback of case study. Although case study simulates complex patient care scenarios, they do not allow students to practice hands-on or experience caregiving emotions. Similarly, the absence of direct patient interactions in case study is a notable limitation. This lack of personal connection and guided practice may hinder the development of caring behaviors. By recognizing these limitations and seeking alternative instructional methods, educational institutions can strive to enhance students' caring skills and equip them with the qualities and behaviors necessary for providing compassionate and patient-centered care.

The findings of this study revealed that neither computer-based simulation nor case study improved students' interest in learning, whereas high-fidelity simulation combined with computer-based simulation was most effective. One possible explanation for the ineffectiveness of computer-based simulation and case study in promoting students' interest is that they may lack the authenticity and immersive nature of real-world experiences [47, 48]. High-fidelity simulation, on the other hand, provides a more lifelike and interactive learning environment, which may enhance students' engagement, interest, and retention [49]. High-fidelity simulation combined with computer-based simulation allows students to interact with the simulation in a hands-on manner while also having access to additional resources and information through computer-based simulation [50]. This combination provides a well-rounded learning experience that can captivate students' attention and keep them engaged. Notably, these findings are exploratory and should be further explored and validated in future studies. Further research should aim to identify the reasons behind the lack of improvement in students' interest in learning when using computer-based simulation and case study alone. Additionally, the impact of different combinations of simulation techniques on students' interest in learning should be investigated to further refine instructional practices.

## Limitations

This study provides valuable insights into the effectiveness of simulation-based education in improving nursing students' competences. However, it is essential to acknowledge and address the study's limitations. One of the limitations is the possible selection bias introduced by the recruiting process. It is possible that students who were more motivated or had a greater interest in simulation-based education may have been more likely to participate in the study. This bias may have influenced the outcomes and interpretation of the results. Additionally, the participants were primarily from one cultural background, which may limit the generalizability of the findings. Future studies should include participants from diverse backgrounds to enhance generalizability. Third, participants assigned to different intervention groups may engage in communication and information sharing, potentially leading to contamination effects. To mitigate this issue, future studies could employ cluster randomized controlled trials, which can effectively minimize the risk of contamination among participants. Finally, the follow-up period was relatively short, which limits the understanding of the long-term impact of simulation-based education on competence. Long-term follow-up studies are needed to evaluate the sustained effect of simulation-based education on competence. Future research should aim to address these limitations to further our understanding of the effects of simulation-based education on undergraduate nursing students' competences.

## Conclusions

The four methods are effective at improving skills and critical thinking both immediately and over time. In addition to high-fidelity simulation, the other three methods promote interprofessional collaboration both immediately and long term. High-fidelity simulation combined with computer-based simulation is the most effective approach for enhancing interest in learning both immediately and long term. Undergraduate nursing students benefit equally from four methods in cultivating their knowledge, interprofessional collaboration, critical thinking, caring, and interest in learning both immediately and over time. High-fidelity simulation and high-fidelity simulation combined with computer-based simulation improve skill more effectively than computer-based simulation in the short term. Nursing educators can select the most suitable teaching method to achieve the intended learning outcomes depending on the specific circumstances.

## Data Availability

The data that support the findings of this study are available from the corresponding author, upon reasonable request.
